# Evaluating the predictability of medical conditions from social media posts

**DOI:** 10.1371/journal.pone.0215476

**Published:** 2019-06-17

**Authors:** Raina M. Merchant, David A. Asch, Patrick Crutchley, Lyle H. Ungar, Sharath C. Guntuku, Johannes C. Eichstaedt, Shawndra Hill, Kevin Padrez, Robert J. Smith, H. Andrew Schwartz

**Affiliations:** 1 Penn Medicine Center for Digital Health, University of Pennsylvania, Philadelphia, Pennsylvania, United States of America; 2 Department of Emergency Medicine, Perelman School of Medicine, University of Pennsylvania, Philadelphia, Pennsylvania, United States of America; 3 Penn Medicine Center for Health Care Innovation, University of Pennsylvania, Philadelphia, Pennsylvania, United States of America; 4 The Center for Health Equity Research and Promotion—Philadelphia Veterans Affairs Medical Center, Philadelphia, Pennsylvania, United States of America; 5 The Wharton School, University of Pennsylvania, Philadelphia, Pennsylvania, United States of America; 6 Positive Psychology Center, University of Pennsylvania, Philadelphia, Pennsylvania, United States of America; 7 Department of Computer and Information Science, University of Pennsylvania, Philadelphia, Pennsylvania, United States of America; 8 Microsoft Research, New York, New York, United States of America; 9 Department of Computer Science, Stony Brook University, Stony Brook, New York, United States of America; University of Oxford, UNITED KINGDOM

## Abstract

We studied whether medical conditions across 21 broad categories were predictable from social media content across approximately 20 million words written by 999 consenting patients. Facebook language significantly improved upon the prediction accuracy of demographic variables for 18 of the 21 disease categories; it was particularly effective at predicting diabetes and mental health conditions including anxiety, depression and psychoses. Social media data are a quantifiable link into the otherwise elusive daily lives of patients, providing an avenue for study and assessment of behavioral and environmental disease risk factors. Analogous to the genome, social media data linked to medical diagnoses can be banked with patients’ consent, and an encoding of social media language can be used as markers of disease risk, serve as a screening tool, and elucidate disease epidemiology. In what we believe to be the first report linking electronic medical record data with social media data from consenting patients, we identified that patients’ Facebook status updates can predict many health conditions, suggesting opportunities to use social media data to determine disease onset or exacerbation and to conduct social media-based health interventions.

## Introduction

Over two billion people regularly share information about their daily lives over social media, often revealing who they are, including their sentiments, personality, demographics, and population behavior. [1–4] Because such content is constantly being created outside the context of health care systems and clinical studies, it can reveal disease markers in patients’ daily lives that are otherwise invisible to clinicians and medical researchers.

Social media content has been shown to contain valuable health signals, though mostly at the population level. For example, Twitter has been used to surveil disease outbreaks[5–7], predict heart disease mortality rates[8], and to monitor public sentiment about health insurance [9]. However, studies that link social media activity and medical diagnoses at the individual level are rare [10] and limited to connections with self-reported mental health over limited samples[11, 12], not validated with health records.

We linked consenting patients’ electronic medical records (EMRs) with their social media data,[13] to ask two novel questions: 1) *Can we predict individuals’ medical diagnoses from language posted on social media*? and 2) *Can we identify specific markers of disease from social media posts*?

**Fig 1:**
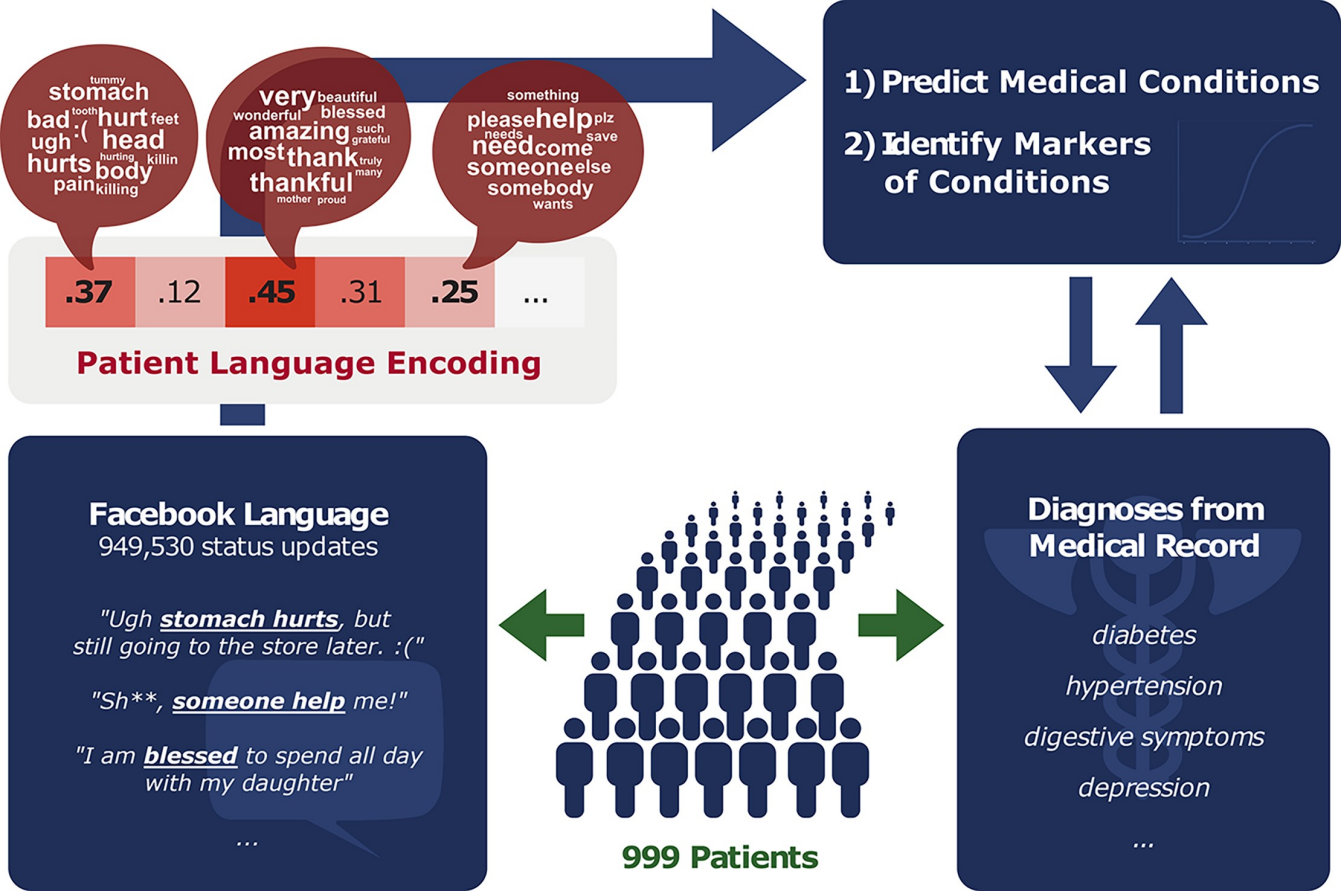
General study design. We extract a *patient language encoding* from the words and phrases within an individual’s Facebook status updates. The three word clouds shown represent the words most prevalent in three example dimensions of the encoding. We then learn predictive models and identify predictive markers for the medical condition categories in the medical records.

Fig 1 depicts our study design and Table 1 lists participant characteristics. We analyzed 949,530 Facebook status updates containing 20,248,122 words across 999 participants whose posts contained at least 500 words. Using natural language processing [14] each participant’s language was encoded as a 700 dimensional *patient language encoding* (i.e., we characterize each user’s social media language by 700 numbers). Diagnoses from participant’s electronic medical records were grouped into 21 categories according to the Elixhauser Comorbidity Index [15] and prevalence within our sample (all categories had at least 30 participants). Data selection procedures are detailed in the supplement (S1 Table).

**Table 1:** Medical condition prevalence and participant characteristics.

Medical condition categories	N
Digestive Abdominal Symptoms	641
Genitourinary Disorders	562
Injury and Poisoning	543
Respiratory Symptoms	433
Pregnancy	323
Skin Disorders	364
Chronic Pulmonary Disease	204
Deficiency Anemia	194
Depression	149
Fluid and Electrolyte Disorders	135
Hypertension	132
Obesity	132
Anxiety	122
Psychoses	73
Drug Abuse	64
Sexually Transmitted Disease	57
Diabetes	49
Blood Loss Anemia	45
Coagulopathy	38
Alcohol Abuse	34
Collagen Vascular Diseases	32
**Participant Characteristics**	**%**
Female sex	76%
**Race or ethnic group**	
Black	71%
White	23%
Asian	2%
Other	4%
**Age categories—year**	
18–23	37%
24–30	33%
31–65	30%

## Materials and methods

We evaluated whether consenting patients’ Facebook posts could be used to predict their diagnoses evident in their electronic medical record (EMR). This study was approved by the University of Pennsylvania Institutional Review Board.

### Data

Participants were drawn from the ongoing Social Mediome study which began recruitment in March 2014 [13]. Adult patients seeking care in an urban academic health system were invited to share their past social media activity and EMR data. Of all participants enrolled through October 2015 and agreeing to share their Facebook data (*N = 1772*), we retrieved status updates up to 5 years prior, ranging from March 2009 through October 2015. We then limited our analyses to those with at least 500 words across all of their Facebook status updates (*N = 999*) as it is found to be a reliable threshold for language analysis of user outcomes. [16]. All data collected from Facebook was from consenting patients and not from anyone in their network, and consistent with the terms of service of the platform.

From the health system’s EMRs, we retrieved demographics (age, sex, and race) and prior diagnoses (by International Classification of Diseases [ICD-9] codes). We grouped ICD-9 codes by Elixhauser Comorbidity Index categories [15],and added categories for medical condition codes not reflected in the index but prevalent in the sample (e.g., pregnancy-related diagnoses) for a total of 41 categories. We then filtered the list of medical condition categories to those attributed to at least 30 patients in the cohort, resulting in 21 categories which are listed in Table 1 along with their ICD-9 codes. Of 1143 patients who shared both social media and EMR data, 999 (87%) had an adequate number of status updates (at least 20).

### Language analysis

We analyzed entire timelines (i.e. all prior posts) of messages posted on Facebook by participants who consented to participate in this study. The medical conditions were extracted from the electronic health records associated with each patient. For the analysis, we set a threshold of >500 words per user and each medical condition had to have at least 30 participants. Our approach was to find topics significantly related to medical conditions through statistical analysis instead of selecting specific posts based on search terms which have high noise. We looked at all posts and did not assign posts as health related or not to avoid this noise.

We extracted all words (unigrams) and word pairs (bigrams: two neighboring words—e.g. “sick of”) from each participant’s status updates. We then grouped similar words into 200 “topics” using Latent Dirichlet Allocation (LDA), a probabilistic technique which automatically clusters words by looking at other words with which they often co-occur [14]. Rather than selecting and grouping words manually, this automatic approach yields topics that may contain slang, misspellings, and variations on contractions which themselves are often predictive [3]. LDA gives us a posterior probability of a topic given a word, *p(topic|word)*, which we use to give each user a topic probability score, *p(topic|participant)*, by multiplying with their probability of mentioning the word:

$$p(participant)=\sum_{word\in topic}p(word)*p(word|participant)$$

**Fig 2:**
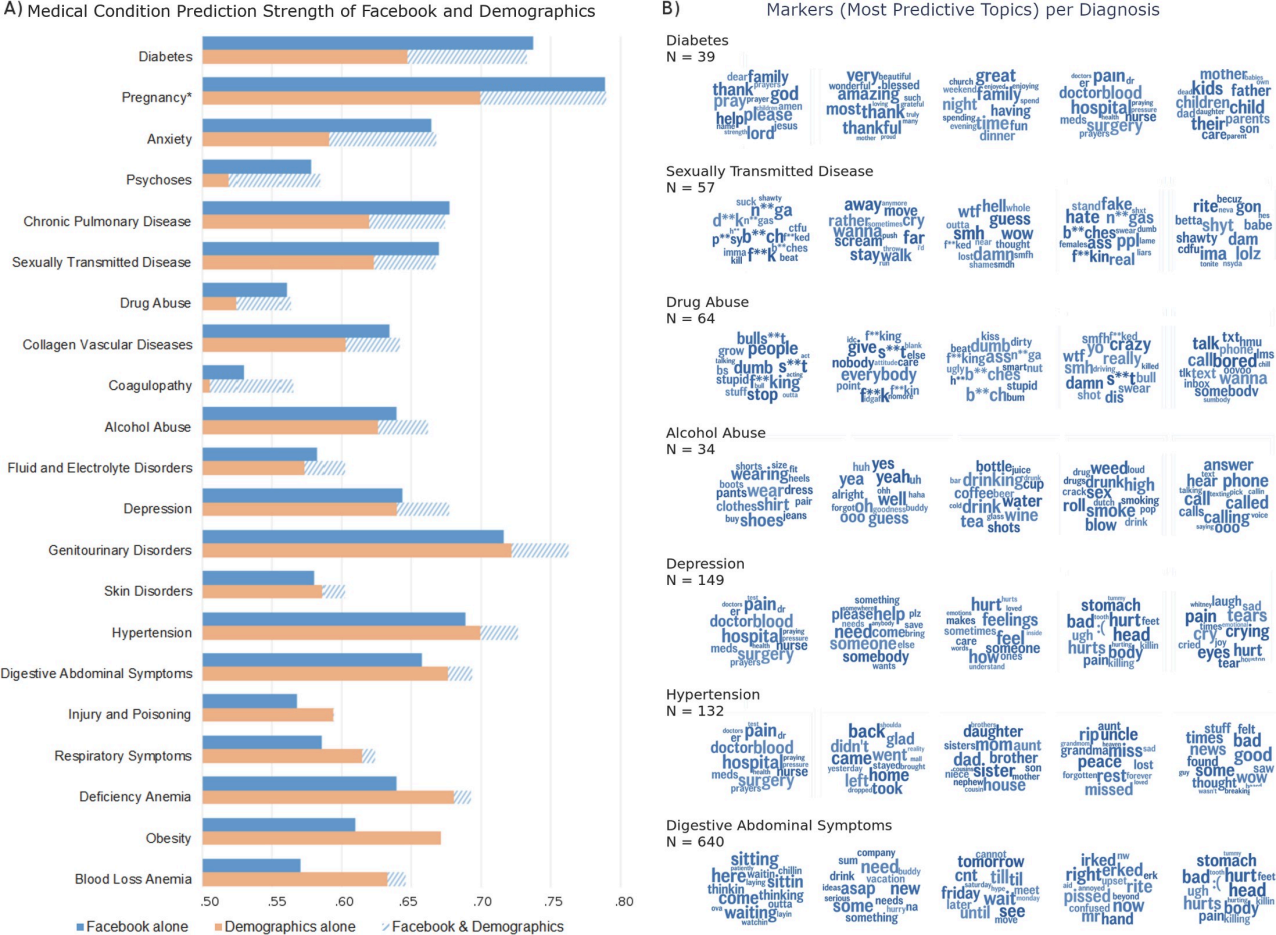
A. Diagnoses Prediction Strength of Demographics and Facebook. This figure represents overall accuracies of Facebook and demographic models at predicting diagnoses. Accuracies were measured using the area under the receiver operating characteristic curve (AUC), a measure of discrimination. The category “Facebook alone” represents predictions based only on Facebook language. “Demographics alone” represents predictions from age, sex, and race. “Demographics & Facebook” represents predictions based on a combination of demographics and Facebook posts. Diagnoses are ordered by the difference in AUC between Facebook alone and demographics alone. For the top 10 categories, Facebook predictions are significantly more accurate than those from demographics (p &lt; .05), and for the top 17 plus iron deficiency anemia, Facebook & demographics are significantly more accurate than Facebook alone (p &lt; .05). * Pregnancy analyses only included females. B. Markers (most predictive topics) per diagnosis. This figure illustrates top markers (clusters of similar words from social media language) most predictive of selected diagnoses categories. Word size within topic represents rank order prevalence in the topic. Expletives were edited and represented by stars (i.e. *). All topics shown, except for those with digestive abdominal symptoms, were individually predictive beyond the demographics (multi-test correct p &lt; .05). (Full results in supplement [S2 Table]).

We generated word clouds to visualize topics using Python (e.g., Fig 2). For each topic, we chose the top 15 words, according to the posterior probability of a word given a topic (*p(word|topic)*), and scaled the size of the word proportionate to its rank (largest implies most prevalent). The shade of the words is randomly jittered in order to improve readability but has no meaning.

While topics provide a coarse-grained set of linguistic features, previous research work in human language analysis has found including individual unigrams and bigrams improves predictive model performance.[1] We included features for the most frequent 20,000 unigrams and bigrams. However, with so many features and only 999 observations, model overfit was a concern [17]. We reduced the word and word pairs to only the top 500 according to their family-wise error rate (*alpha*) with the given medical conditions using only the training portion within cross-validation (see *10-fold cross-validation below*). These 500 word and word pair features with the 200 topics formed our 700 dimensional *patient language encoding*.

### Predicting diagnoses

For each medical condition, we built three predictive models associating Facebook posts with EMR-based diagnoses: Model 1 used Facebook language (unigrams, bigrams, and topics). Model 2 used the demographics of age, sex, and race. Model 3 used both demographics and Facebook language. For each model, we used statistical learning techniques from the field of machine learning [17]. For model 1, which included hundreds of predictors, we used extremely random trees (ERT) [18], variant of random forests which are well suited to handle many predictors. Within each ERT, we used 1,000 estimators with a Gini coefficient as criteria for split points. For model 2, we fit an L2-penalized (ridge) logistic regression, an ideal model when there are relatively few predictors (to confirm, we also ran the ERT approach and found all accuracies were the same or lower). Model 3 was an ensemble of models 1 and 2 created by an average of the predicted probabilities from each model, weighted by the AUC of the model over the training set

$$P_{ensemble}(Facebook,Demographics)=\frac{AUC1\;(PERT\;(Facebook))+AUC2\;(Plog-ref\;(Demographics))}{AUC1+AUC2}$$

where AUC_1_ and AUC_2_ correspond respectively to the training set AUCs for models (1) and (2).

This approach compares the predictive ability of Facebook language (*model 1*) to that of demographics (*model 2*) as well as the incremental contribution of Facebook language to demographics (comparing *model 3* to *model 2*). We measured predictive ability using the area under the receiver operating characteristic curve (AUC), a measure of discrimination. A value of 0.5 is expected due to chance; a value of 1 indicates perfect prediction. To evaluate controlling for model overfit, we measured AUC *with 10-fold cross-validation* [17]: we split our sample into 10 equal-sized, non-overlapping, and stratified partitions, fit models over 9 partitions (including the selection of the 500 unigram and bigram features), and tested the fit model on the remaining held-out partition. This process repeats ten iterations such that each partition is used as a the held-out test partition once. We then used a Monte Carlo permutation test [19]with 100,000 iterations to calculate significance of the difference between any two AUCs, correcting for multiple hypothesis testing using the Benjamini-Hochberg False-discovery rate procedure [20]. Testing out of sample as facilitated from 10-fold cross-validation and correcting for multiple tests was key to insuring a rigorous and fair evaluation of predictive accuracies.

Prediction accuracy was evaluated using the area under the receiver operating characteristic curve (AUC), a measure of discrimination in which a value of 0.5 is expected due to chance, 0.6 is considered moderate, and above 0.7 is considered a strong prediction from a behavior [17, 18]. Most variables associated with medical conditions tend to have AUCs which fall between .50 and .85 [19], but often include variables derived from invasive tests. As a reference [19], within our sample, body-mass index predicted diabetes with an AUC of .64. Holding to this standard, we consider an AUC of 0.65 and above as strong.

### Identifying medical condition markers

Our approach to identifying medical condition markers was similar to that of predicting diagnoses but rather than including all language variables in a model, we considered only one topic at a time. We evaluated each individual topic’s predictive ability by comparing three AUCs: 1) from usage scores for the topic alone, 2) from a logistic regression model [20] over age, sex, and race, and 3) from a logistic regression model over age, sex, race, plus the topic. In this way, we focus simply on one topic at a time in order to illuminate its relationship with the medical condition categories. AUCs were determined out-of-sample when using multiple predictors (i.e. 2 and 3) and a permutation test was used to assess significance of individual topics. We used word clouds to display topics—visual depictions of the most prevalent words in each topic where each word’s size corresponds to its contribution.

We also tested whether we could empirically identify individual markers of diagnoses in the daily social media language of patients. To do this, we considered a portion of the 700 dimensional language encoding which includes 200 topics [14], or sets of similar words (e.g. pain, hospital, blood). We then followed a similar approach as our overall predictive analysis, except treating each individual topic as a potential marker and building models from: 1) usage scores for the topic alone, 2) demographics, and 3) both demographics and the topic. AUCs were determined out-of-sample [20] when using multiple predictors (i.e. cases 1 and 3) and a permutation test [21] was used to assess significance of individual topics along with the Benjamini-Hochberg procedure to control for false discoveries [22]. Further details are provided in the supplement (S1 Text).

In order to explain the predictive ability of a topic in other terms, we listed a couple examples of the increase in likelihood between participants in the top quartile of mentioning a topic and those in the bottom quartile of mentioning the same topic. For example, considering mental health conditions, patients in the top quartile of mentioning the *want wanted give ask* topic were 4.1 times (95% CI: [1.3, 26.6]) more likely to have been diagnosed with psychoses than those in the bottom quartile of mentioning that same topic. For these calculations, we used maximum likelihood estimates for the mean probability that one had the disease in both the top and bottom quartile and then divided the two probability estimates to get the multiple (e.g. 4.1 times). Finally, we used a bootstrap resampling procedure [20] with 10,000 iterations to calculate 95% confidence intervals for the differences in likelihoods.

All statistical analyses were performed in Python and the exact code used is available as part of the Differential Language Analysis ToolKit (http://dlatk.wwbp.org).

## Results and discussion

We identified that: 1) all 21 medical condition categories were predictable from Facebook language beyond chance (multi-test corrected *p <* .*05*), 2) 18 categories were better predicted from a combination of demographics and Facebook language than by demographics alone (multi-test corrected *p <* .*05*), and 3) 10 categories were better predicted by Facebook language than by the standard demographic factors (age, sex, and race). These results are depicted in Fig 2 which shows the accuracies of the three predictive models across all 21 diagnoses categories.

The medical condition categories for which Facebook statuses show the largest prediction accuracy gains over demographics include diabetes (*AUC =* .*74*), pregnancy (*AUC =* .*79*; females only) and the mental health categories anxiety (*AUC =* .*66*), psychoses (*AUC =* .*58*) and depression (*AUC =* .*64*).

Fig 2 depicts the individual language markers of diagnoses for selected categories that showed the highest predictive power over and above demographics (*p <* .*05*). Many topic markers of diagnoses reveal characteristic behavior or symptoms. For example, alcohol abuse was marked by a topic mentioning *drink*, *drunk*, *bottle*. Topics expressing hostility (e.g. *people*, *dumb*, bulls**t, b**ches) were the predominant marker of *drug abuse* and also marked psychoses. Topics most associated with depression suggested somatization (e.g. *stomach*, *head*, *hurt*) and emotional distress (e.g. *pain*, *crying*, *tears)*. Other markers may suggest socio-environmental variables associated with disease risk; for example, diabetes was predicted by religious language (e.g. *god*, *family*, *pray*) even when controlling for demographic variables. This does not mean that everyone mentioning these topics has the condition, but just that those mentioning it are more likely to have it. For example, the top 25% of patients mentioning the (*god*, *family*, *pray*) topic were 15 times (95% CI: [3.16, inf]) more likely to have been diagnosed with diabetes than those in the bottom 25% of mentioning that same topic. This association may be specific to our patient cohort and suggests the potential to explore the role of religion in diabetes management or control.[21–23].

**Fig 3:**
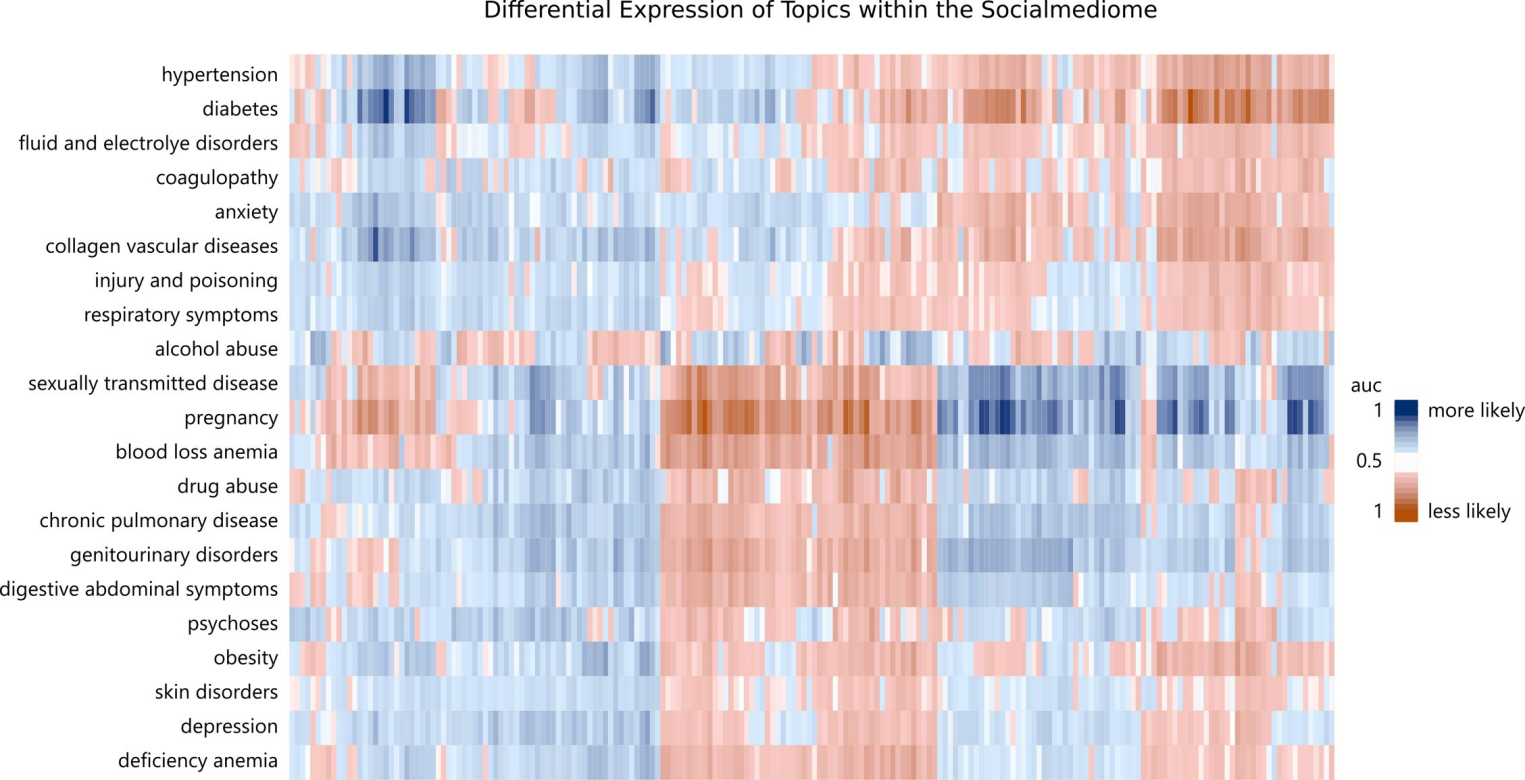
Differential expression of topics across medical conditions within the social mediome. Analogous to studying the differential expression of a genome, topics of the social mediome can be explored differentially across diagnoses. The 21 rows represent all medical condition categories of the study ordered using hierarchical clustering while the 200 columns indicate the predictive strength[24] (measure by *area under the ROC curve*) of each potential language marker (topics). Blue topics were more likely to be used by patients with the given medical condition and orange topics were less likely to be mentioned. Medical condition categories each have unique patterns of markers. These encodings allow for the prediction of diagnoses and identification of diagnoses with similar patterns of markers.

Just as contemporary biobanking aims to collect biosamples that reflect the genome and to link individual genetic information to phenotypic manifestations of health, social media data can be conceived as a “social mediome” whose individual expression can also be banked in a registry and linked to more phenotypic markers of health and disease [10]. Similar to visualizations of gene expression, as depicted in Fig 3, patterns of language can be associated with diagnoses to reveal similarities and difference between diagnoses.

Like genomic data banking, the power of social media language to predict diagnoses raises parallel questions about privacy, informed consent, and data ownership. Social media data banking has the advantage that physical biospecimens do not need to be collected—access and consent can occur electronically and remotely, for example by sharing a Twitter handle or one-click authorization of a Facebook application. The extra ease with which social media access can be obtained creates extra obligations to ensure that consent for this kind of use is understood and intended. Efforts are needed to ensure users are informed about how their data can be used, and how they can recall such data. At the same time, such privacy concerns should be understood in the context of existing health privacy risks. It is doubtful that social media users fully understand the extent to which their health is already revealed through activities captured digitally[25]. For example, applications to use social media data-derived risk profiles to price car insurance have already been tested [26].

We found that the language people use in Facebook is predictive of their health conditions reported in an EMR, often more so than typically available demographic data. Although some early research has linked social media language use with health [11, 12, 27] this is the first study to the best of our understanding to do so at the level of the patient with EMR data.

Social media information has the advantage that it often has a built in communication channel back to patients. For example, Facebook now allows users to flag posts within their network that they think may suggest suicidal ideation. Facebook then anonymously provides resources for individuals at risk. A similar patient-centered approach could be applied to a broader set of conditions allowing individuals and their networks (for those who opt-in) to have early insights about their health-related digital footprints. These considerations reveal simultaneous promise and challenge in banking and mining the social mediome and echo similar debates that have arisen around use of the human genome, including logistical and ethical challenges with recontact and communicating risk to patients as predictive ability expands, often in unanticipated ways.

This study has several limitations. Constellations of predictive words often do not represent causal mechanisms and the findings are correlational. However, in revealing what people think, feel, and do, social media patterns capture emotional, cognitive, behavioral and environmental markers that have substantial predictive validity and are otherwise fairly elusive to researchers and clinicians. We equally weighted recent and remote posts in our analyses; adjustment for recency might reveal different or stronger associations. Also, predictive associations of language with disease may vary across populations, requiring rederivation of language markers in different sub-populations that may point to specific, ecologically-appropriate considerations. Further, we utilized logistic regression and extremely randomized trees as modeling algorithms for their interpretability and simplicity considering the number of samples in our study. Prior work has demonstrated, transfer learning from pre-trained text-based multi-layer neural network architectures could lead to higher predictive performance [28]. Also, the participants in this study represented a convenience sample (of primarily African American women) who were receiving care at an urban academic medical center, and not representative of the general population. Prior work has shown that users vary in the amount and diversity of self-representation across different online platforms[16, 29]. Future studies could compare difference in health related information disclosed by users of different demographic populations and on other social media platforms (e.g. Twitter).

Social media, like genomic information, offers enormous promise to personalize health care. This work is complementary to a growing body of literature using big data analytics for EMR data [30–32] and provides new insights for applying machine learning to find signal about health in non-healthcare generated data (e.g. social media).

People’s personality, mental state, and health behaviors are all reflected in their social media and all have tremendous impact on health. This is the first study to show that language on Facebook can predict diagnoses within people’s health record, revealing new opportunities to personalize care and understand how patients’ ordinary daily lives relate to their health.

## Supporting information

S1 Text Methodologic details. This text includes further explanations about methodologic approaches.
(DOCX)

S1 Table Elixhauser and additional categories of ICD codes used for each health condition (s). This Table includes the categories used to define each health condition.
(DOCX)

S2 Table Topics and predictive values for each medical condition. This table includes a comprehensive list of all topics and corresponding predictive values (AUC) with demographics, and each medical condition category from our study cohort.
(DOC)
