# Supplementary material for: Evaluating the predictability of medical conditions from social media posts
Source: PLoS One. 2019 Jun 17;14(6):e0215476. doi: 10.1371/journal.pone.0215476 (PMC6576767; doi:10.1371/journal.pone.0215476)
Supplement: S1 Text — This text includes further explanations about methodologic approaches. (DOCX) [file pone.0215476.s001.docx]

**S1 Text**

MINIMUM WORD THRESHOLD FOR INCLUSION

The minimum thresholds were selected based on prior literature. Kern et al., (2016) established that error of a word-based predictive model flattens out approximately between 500 and 1000 word minimum.[1] Our number of subjects were quite limited (999 as opposed to the 50,000 in Kern et al.), so we chose the lower threshold to be most inclusive here. Schwartz et al. (2013) and Jaidka et al. (2018) explore power as a function of number of subjects, finding significant associations between words and human-level traits and states begin appearing at approximately 60 subjects (or 30 positive and 30 negative subjects when the outcome is dichotomous as it was here).[2 3]

WORD AND TOPIC EXTRACTION.

We automatically extracted all word and word pairs (*bigrams* -- two neighboring words) using a modern tokenizer, a computer program which splits sentences or phrases into individual word or word-like elements such as punctuation, emoticons (e.g. ‘<3’ to represent a heart, ‘:-)’ to represent a smile) or abbreviations (e.g., ‘omg’ to represent *oh my gosh* and ‘lol’ to represent *laugh out loud*). For each participant, we recorded individual word and word pair features as their relative frequency: the percentage of the total number of words the participant wrote. For example, the word ‘the’ often makes up 2.2% of the words people use on Facebook. We removed all features mentioned by less than 5% of users (Kern et al., 2016).

We also extracted “topics” -- groups of similar words -- using Latent Dirichlet Allocation (LDA). The LDA probabilistic model assumes that documents (here, Facebook status updates) contain a distribution of topics which then contain a distribution of words. Ultimately, words are grouped together by considering the other words they appear with. We use the Mallet package implementation of the LDA to cluster the status updates of all participants. Mallet gives us a posterior probability of a word being generated from a topic, *p(topic|word)*, which we use to give each user a topic probability score, *p(topic|participant)*, by multiplying with their probability of mentioning the word:


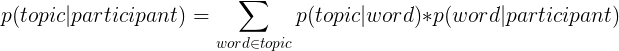


We also generated word clouds to visualize topics using Python. Based on the posterior probability of a word given a topic (*p(word|topic)*), we chose the 15 most prevalent words for the topic and scaled the size of the word proportionate to its rank (largest implies most prevalent). The shade of the words is randomly jittered in order to improve readability but has no meaning. Kern et al. (2016) notes a tradeoff between clarity of topics and the amount of duplicates.[1] Thus, we explored 50, 100, 200, and 500, finding that 200 yielded both qualitatively clear topics but without a lot of overlap.

FULL PREDICTIVE MODELS. Our predictive modeling fit 3 models per diagnosis category: (1) one based on Facebook alone, (2) one based on demographics alone (sex, race, and age –the latter in three terciles to capture nonlinear relationships), and (3) a combination of (1) and (2). For the model based on Facebook alone (1), we used all topics and 1 to 2-word sequences (derived from the tokenization approach mentioned above) as possible features. This resulted in 20,090 features and, with only 999 observations, model overfit is a concern.[4] Therefore, we fed these to a feature selection to reduce only to those features with some small relation to the disease diagnosis, evidenced by a family-wise error rate (*alpha*) of *10* (i.e. the p-value multiplied by the total number of features considered should have been less than 10). Using this reduced feature set, we then fit Facebook model using *extremely random trees (ERT)* containing 1,000 trees utilizing a gini coefficient to choose split points*.* Model (2), which only had 5 features, used logistic regression with L2 regularization. Theoretically, *ERT* is better suited to many noisy features while logistic regression is better suited to capture variance. Still, we tried the *ERT* classifier for model (2) and verified all results were either the same or worse than as with logistic regression. Finally, model (3) was an ensemble of models (1) and (2) created by an average of the predicted probabilities from each model, weighted by the AUC of the model over the training set:


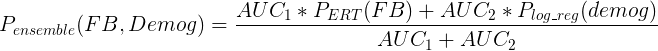


where AUC_1_ and AUC_2_ correspond respectively to the training set AUCs for models (1) and (2).

Three accuracies, as AUCs, were established corresponding to the three models. To control for overfit in the evaluation,[4] all models were fit (including the feature selection step) over “training data” and evaluated over held-out “test data” via a 10-fold cross-validation procedure using stratified folds. For each diagnosis category, subjects were divided randomly into 10 subsets preserving the percentage of true diagnoses per fold. AUCs for each of the 3 models were then calculated across the results of all folds at once. P-values were calculated using a permutation test: for each AUC, the diagnosis was shuffled relative to language and controls over 100,000 iterations to generate a null distribution of coefficients. All p-values were corrected for multiple hypotheses (i.e. one for each diagnosis) using the Benjamini-Hochberg False-discovery rate procedure.[5]

INDIVIDUAL TOPIC PREDICTIVE MODELS.

In order to explain the predictive ability of a topic in other terms, we listed a couple examples of the increase in likelihood between participants in the top quartile of mentioning a topic and those in the bottom tercile of mentioning the same topic. For example, considering mental health conditions, patients in the top quartile of mentioning the:  *want wanted give ask* topic were 4.1 times (95% CI: [1.267 26.58) more likely to have been diagnosed with psychoses than those in the bottom quartile of mentioning that same topic. For these calculations, we used maximum likelihood estimates for the mean probability that one had the disease in both the top and bottom quartile and then divided the two probability estimates to get the multiple (e.g. 4.1). Finally, we used a bootstrap resampling procedure[cite], with 10,000 iterations to calculate 95% confidence intervals.[6]

DISEASE CLUSTERING BY TOPICS

To find diseases with similar topic profiles, we used aglomerative clustering based on dissimilarity of topic correlations between each diagnosis category. Specifically, we use a *complete linkage* method where by the proximity between two clusters is the proximity between their two most distant objects. In other words, the two most distant clusters from other members cannot be much more dissimilar than other quite dissimilar pairs (as in a circle). Such hierarchical clusters are compact in that the joining of two groups at each level insures none of its elements are very far from each other.

Figure S1: Select 5 topics most predictive of each disease, positively and negatively. Red boxes indicate the topics is significantly predictive beyond age, gender, and race. All topics shown are predictive beyond chance. AUCs for the topic alone and topic + demographics are provided below each topic as well as any demographic factor that is positively related to the topic. (Figure attached)

References

1. Kern ML, Park G, Eichstaedt JC, et al. Gaining insights from social media language: Methodologies and challenges. Psychol Methods 2016;**21**(4):507-25 doi: 10.1037/met0000091[published Online First: Epub Date]|.

2. Schwartz HA, Eichstaedt JC, Kern ML, et al. Personality, gender, and age in the language of social media: the open-vocabulary approach. PloS one 2013;**8**(9):e73791 doi: 10.1371/journal.pone.0073791[published Online First: Epub Date]|.

3. Jaidka K, Guntuku S, A. SH, Buffone A, Ungar L. Facebook versus. Twitter: Differences in self-disclosure and trait prediction. Proceedings of the International AAAI Conference on Web and Social Media 2018 2018

4. Hastie T TR, Friedman J, Tibshirani R. . *The elements of statistical learning: data minin, inference, and prediction*: Springer New York, 2009.

5. Benjamini Y, Hocberg Y. Controlling the False Discovery Rate: A Practical and Powerful Approach to Multiple Testing. Journal of the Royal Statistical Society. Series B (Methodological) 1995;**57**(1):289-300

6. Efron B, Tibshirani RJ. *An Introduction to the bootstrap*: Chapman and Hall/CRC, 1993.
