# Supplementary material for: Evaluating the predictability of medical conditions from social media posts
Source: PLoS One. 2019 Jun 17;14(6):e0215476. doi: 10.1371/journal.pone.0215476 (PMC6576767; doi:10.1371/journal.pone.0215476)
Supplement: S1 Table — This Table includes the categories used to define each health condition. (DOCX) [file pone.0215476.s002.docx]

**S1 Table. Elixhauser and additional categories of ICD codes used for each health condition(s)**

| **Health condition (s)** | **Cluster N** | **Top 20 ICD codes (N)** |
| --- | --- | --- |
| Digestive Abdominal Symptoms | 641 | 789.09 (361), 789.00 (328), 787.02 (241), 787.01 (239), 787.91 (155), 787.03 (110), 789.06 (84), 787.20 (74), 789.03 (57), 789.04  (42), 789.01 (40), 787.30 (37), 789.07 (25), 787.10 (19), 789.02 (19), 787.99 (14), 789.39 (11), 789.30 (7), 789.59 (7), 787.60 (6) |
| Genitourinary Disorders | 562 | 599.00 (221), 623.50 (156), 616.10 (152), 623.80 (144), 625.90 (99), 616.00 (66), 626.80 (60), 620.20 (55), 626.20 (50), 590.80  (47), 599.70 (45), 626.00 (43), 626.40 (42), 614.90 (39), 611.72 (38), 625.80 (38), 584.90 (36), 625.30 (34), 611.71 (27), 626.90  (23) |
| Injury And Poisoning | 543 | 845.00 (56), 959.01 (38), 847.00 (36), 995.30 (36), 959.80 (35), 959.70 (34), 883.00 (30), 995.91 (30), 848.80 (26), 998.59 (23),  840.90 (21), 844.90 (19), 846.90 (19), 847.90 (19), 920.00 (19), 924.11 (19), 847.20 (15), 995.27 (15), 850.90 (14), 959.09 (14) |
| Respiratory Symptoms | 433 | 786.20 (225), 786.59 (208), 786.05 (173), 786.50 (155), 786.09 (57), 786.07 (49), 786.52 (46), 786.51 (16), 786.30 (13), 786.40 (9),  786.06 (4), 786.60 (4), 786.90 (4), 786.39 (3), 786.01 (2), 786.00 (1), 786.02 (1), 786.03 (1), 786.10 (1), 786.70 (1) |
| Skin Disorders | 364 | 695.90 (86), 692.90 (70), 698.90 (70), 698.10 (44), 682.20 (40), 682.30 (31), 682.60 (29), 682.90 (24), 682.00 (21), 706.10 (21),  708.90 (21), 698.80 (16), 704.80 (16), 682.50 (15), 709.90 (15), 709.80 (12), 686.90 (10), 705.83 (10), 706.20 (10), 709.20 (9) |
| Pregnancy | 323 | V27.0 (257), V22.1 (231), V22.2 (186), V24.2 (115), V22.0 (82), 650.00 (69), V24.0 (22), V24.1 (8), 651.03 (7), V27.2 (7), 651.01 (6),  651.00 (4), 651.10 (1), 651.11 (1), 651.13 (1), V27.5 (1) |
| Chronic Pulmonary Disease | 204 | 493.90 (167), 493.92 (54), 490.00 (23), 493.00 (5), 493.81 (4), 496.00 (4), 491.21 (2), 492.80 (2), 493.20 (2), 493.82 (2), 494.10  (2), 491.00 (1), 491.22 (1), 492.00 (1), 494.00 (1), 495.90 (1) |
| Deficiency Anemia | 194 | 285.90 (168), 280.90 (72), 280.80 (2), 281.90 (2), 281.10 (1) |
| Depression | 149 | 311.00 (138), 300.40 (28), 309.00 (5), 301.12 (2) |
| Fluid And Electrolyte Disorders | 135 | 276.51 (94), 276.80 (38), 276.10 (26), 276.20 (19), 276.70 (17), 276.52 (14), 276.69 (12), 276.60 (5), 276.90 (5), 276.50 (4), 276.00  (3), 276.30 (2), 276.40 (1) |
| Hypertension | 132 | 278.00 (132) |
| Obesity | 132 | 401.90 (110), 401.10 (64), 642.00 (14) |
| Anxiety | 122 | 300.00 (107), 300.01 (20), 300.02 (18), 300.09 (2) |
| Psychoses | 73 | 296.80 (23), 296.90 (22), 296.20 (18), 296.32 (11), 296.89 (9), 296.30 (8), 296.33 (7), 296.35 (6), 296.70 (5), 296.31 (4), 296.50  (4), 298.90 (4), 295.90 (3), 296.00 (2), 296.22 (2), 296.25 (2), 296.36 (2), 296.52 (2), 297.90 (2), 298.80 (2) |
| Drug Abuse | 64 | 305.20 (36), 305.90 (22), 305.50 (7), 305.60 (6), 292.00 (5), 304.01 (5), 304.00 (4), 304.30 (3), 304.90 (3), 305.23 (3), 304.20 (2),  304.61 (2), 305.40 (2), 305.63 (2), 292.85 (1), 304.03 (1), 304.10 (1), 304.11 (1), 304.21 (1), 304.31 (1) |
| Sexually transmitted disease | 57 | 99.90 (34), 98.00 (12), 99.80 (7), 97.90 (5), 99.30 (2), 91.20 (1), 98.50 (1), 98.51 (1), 98.86 (1), 98.89 (1), 99.50 (1), 99.53 (1),  99.55 (1), 99.59 (1) |
| Diabetes | 49 | 250.00 (45), 250.02 (13), 250.01 (8), 250.10 (5), 250.13 (5), 648.00 (4), 250.03 (3), 250.12 (1), 250.20 (1) |
| Blood loss anemia | 45 | 648.20 (31), 280.00 (15) |
| Coagulopathy | 38 | 287.50 (25), 286.40 (6), 287.49 (6), 286.30 (3), 286.90 (3), 287.31 (2), 286.00 (1), 286.20 (1), 286.60 (1), 286.70 (1), 287.10 (1),  287.30 (1), 287.40 (1) |
| Alcohol abuse | 34 | 305.00 (17), 303.00 (14), 303.90 (3), 303.91 (1), 303.93 (1) |
| Collagen vascular diseases | 32 | 710.00 (15), 714.00 (8), 720.20 (6), 714.90 (5), 710.90 (4), 701.00 (2), 710.10 (2), 710.20 (1), 714.30 (1), 714.81 (1), 720.00 (1) |
