# Supplementary material for: Evaluating the predictability of medical conditions from social media posts
Source: PLoS One. 2019 Jun 17;14(6):e0215476. doi: 10.1371/journal.pone.0215476 (PMC6576767; doi:10.1371/journal.pone.0215476)
Supplement: S2 Table — This table includes a comprehensive list of all topics and corresponding predictive values (AUC) with demographics, and each medical condition category from our study cohort. (DOC) [file pone.0215476.s003.doc]

Topics	Demographics	Outcomes and topic AUCs


Num	Clouds	Sex         Age     Race	Digest      Genit        Injur        Resp        Preg         Skin        Pulm        Def
Anem

Depres      Fluid      Hypert      Obes         Anx        Psych       Drug        STD        Diab        BL
Anem

Coag Alc Collag


0	♂ 0.71      ↑ 0.63      W 0.83	0.54           0.52         −0.61       −0.60         0.64         −0.56     −0.52      0.63      −0.59       −0.51       −0.56       −0.64         0.57      −0.54       −0.54     −0.72     −0.55       −0.51       −0.54       −0.51      −0.62
1	♀ 0.57       ↓ 0.63       B 0.76	0.72      −0.58         0.56        0.52         −0.64      0.51       0.53      −0.69      0.54          0.60       0.53        0.57         −0.53     −0.53         0.51       0.67      −0.50        −0.50     −0.61     −0.57	0.54
2	↓ 0.70       B 0.79	0.64       −0.56         0.56        0.53         −0.64     −0.54        −0.51        −0.62          0.52           0.66       −0.52          0.57         −0.54     −0.52        −0.53         0.69        0.62       −0.50    −0.58       −0.58	0.61
3	♀ 0.61       ↓ 0.74       B 0.74	−0.59     −0.51      0.55       0.55      −0.62     −0.55      0.55      −0.65         0.55          0.53      −0.62      0.60      −0.54     −0.52       −0.52         0.68       0.60      −0.51       −0.59      0.59	0.60
4	♂ 0.60      ↑ 0.68      W 0.67	0.70          0.56         −0.53       −0.54      0.50      −0.56         0.58          0.52      −0.54     −0.51         0.55        −0.57      0.53          0.52         −0.53       −0.67      0.60          0.54          0.54       0.56   −0.53
5	↓ 0.61       B 0.77	0.58      −0.59      0.56       0.60      −0.60      0.53       0.52      −0.55         0.53          0.56      −0.53         0.61      −0.55      0.52         −0.53         0.72       0.57        0.51      −0.57      0.52	0.63
6	♀ 0.74       ↑ 0.57       B 0.65	−0.60         0.57       0.56        0.50           0.63          0.60       0.60       0.55        0.60          0.54       0.54        0.62          0.60        0.53          0.63       0.59        0.64           0.52   0.54          0.54	0.53
7	♀ 0.63       5 0.56       B 0.59	−0.60         0.51       0.55       0.55       0.52       0.55       0.59      −0.58         0.56          0.53       0.52        0.59           0.60           0.50           0.51           0.57          0.53        0.53     0.54       0.51	0.55
8	♂ 0.56       ↑ 0.66      W 0.79	0.72           0.55       −0.54       −0.53      0.54      −0.58      0.54       0.51       −0.53         0.66           0.51       −0.55         0.51        0.52           0.55         −0.66      0.55        0.51    0.59       −0.56        −0.57
9	♀ 0.55      5 0.55      W 0.64	0.53       0.51      −0.54       −0.59       −0.69       −0.56       −0.59         0.62        0.51         −0.61     −0.61         0.56          −0.57       −0.57       −0.54       −0.56       −0.58         0.51           0.69         −0.58       −0.51
10	↓ 0.79       B 0.78	0.69      −0.51      0.58       0.54      −0.57     −0.57     −0.57     −0.64         0.51          0.54      −0.57      0.58      −0.58     −0.52       −0.53      0.66      −0.59     −0.53       −0.63    −0.56	0.62
11	♀ 0.55      ↑ 0.61      W 0.70	−0.52       −0.54       −0.53       −0.52        0.54      −0.51       −0.52        0.64     −0.51      0.66          0.53        −0.54      0.56         −0.51       −0.56       −0.59       −0.59       −0.51         0.67        −0.57       −0.56
12	♀ 0.60       ↑ 0.77      W 0.63	−0.59         0.51         −0.52         0.57           0.65           0.54           0.60           0.62        0.50           0.56           0.55        0.55        0.58        0.51           0.57         −0.60        −0.52         0.51           0.58           0.54      −0.57
13	♀ 0.58       ↓ 0.63       B 0.69	−0.61        −0.50         0.53        0.57        0.66        0.57           0.51      −0.57       0.51          −0.52        −0.51          0.57          −0.54        −0.57        −0.50         0.61      −0.56       0.52   −0.50         0.50	0.60
14	5 0.55       B 0.68	0.51       0.54       0.52       0.58      −0.55         0.52          0.54       0.58        0.52          0.52      −0.53      0.56         −0.61         0.55           0.52          0.59       0.55        0.54       0.51       0.50	0.54
15	♀ 0.65	W 0.62	−0.55         0.59           0.70           0.58        0.65           0.60           0.52       −0.61         0.52           0.66         −0.56         0.55           0.54       −0.52         0.50      −0.54      0.50       −0.53   0.61       0.50	0.69
16	B 0.72	0.73          0.56       0.50       0.58       0.56       0.52       0.55       0.63       0.55          0.53       0.60       0.59       0.57       0.53          0.51       0.60          0.63       0.50      0.67       0.50	0.56
17	♀ 0.68       5 0.57       B 0.63	−0.55       −0.61        0.53       0.58      −0.67        0.55       0.56       0.72       0.60          0.57       0.52       0.64       0.53       0.57          0.55       0.61       0.59       0.53         0.58      0.56	0.60
18	↑ 0.74       B 0.60	0.70          0.50        0.67          0.52       0.52        0.58           0.51          0.66       0.52        0.57          0.56       0.51       0.61        0.56           0.57         −0.51      0.61           0.59          0.61        0.53   −0.60

19	♀ 0.57	W 0.78	−0.52         0.55         −0.56       −0.54       −0.65       −0.54       −0.53        0.63      −0.54     −0.53        0.57      −0.55     −0.50       −0.51       −0.52      −0.67     −0.56       −0.50       −0.62       −0.54       −0.63
20	♂ 0.59      5 0.55	0.68           0.59           0.66         −0.59      0.61         −0.53       −0.58       −0.62         0.54           0.63         −0.56         0.55           0.58           0.53         −0.50         0.56           0.61           0.54         −0.60       −0.56  0.64
21	5 0.56       B 0.74	0.68          0.55       0.57       0.63       0.53       0.57       0.56        0.61           0.55           0.65           0.58           0.61           0.55           0.54           0.51          0.62       0.56        0.57   0.55       0.54	0.53
22	↑ 0.54       B 0.57	−0.54      0.53       0.51       0.56       0.58        0.50          0.54       0.54        0.58           0.66           0.55           0.51         −0.59         0.53          0.51        0.55          0.52        0.54    0.52       0.51	0.52

23	↑ 0.56     W 0.73	0.58       0.60         −0.52       −0.55       −0.62     −0.53       −0.57        0.66      −0.56     −0.55        0.50      −0.57      0.54      −0.52        0.53      −0.62       −0.54       −0.54        0.61      −0.50     −0.61

24	♀ 0.56       ↑ 0.78       B 0.55	0.70          0.52      −0.62         0.51       0.53        0.58           0.55          0.68        0.55          0.52       0.57       0.53       0.65        0.57           0.60         −0.53      0.61           0.57          0.59        0.53    −0.57
25	♀ 0.66      ↓ 0.63	−0.55       −0.55        0.53       0.54         −0.65         0.55          0.59      −0.55      0.56          0.52      −0.55      0.56         −0.57       −0.55       −0.51        0.60      −0.52       −0.50       −0.67      0.57      0.52
26	♂ 0.77      ↑ 0.59      W 0.66	0.54      −0.56       −0.53       −0.56         0.51      −0.56       −0.50      0.63      −0.58     −0.58         0.60         −0.61      0.55         −0.55       −0.54       −0.62     −0.53       −0.51       −0.51       −0.51      −0.52
27	↓ 0.67       B 0.82	0.59      −0.53         0.59       0.58      −0.61      0.57       0.53      −0.62         0.53          0.51      −0.50         0.60      −0.55        −0.57        −0.54         0.73       0.58      −0.55       −0.50      0.52	0.66
28	B 0.70	0.64         −0.59      0.51       0.56       0.57       0.50       0.50      −0.62      0.50           0.67         −0.52         0.54         −0.61         0.53           0.54           0.58          0.53        0.53         −0.58         0.51	0.57
29	↑ 0.66	0.68          0.54       0.66          0.53       0.58          0.51       0.52       0.58       0.51          0.51       0.52       0.51          0.54       0.52          0.51       0.56       0.54       0.53          0.58          0.51      −0.61
30	♀ 0.67      ↓ 0.72      B 0.71	−0.50       −0.56        0.51       0.50         −0.51         0.60          0.51      −0.64         0.55          0.62      −0.53        0.60      −0.60       −0.52       −0.54      0.66      −0.53       −0.53     −0.57       −0.55	0.55
31	↓ 0.68       B 0.62	0.69      −0.51      0.66        0.53       −0.55       −0.58     −0.57     −0.51         0.52         −0.65     −0.55       0.56         −0.53     −0.51       −0.52       0.53           0.57       −0.53   −0.70     −0.52	0.55
32	↓ 0.69       B 0.81	0.61       −0.57         0.58       0.54       −0.51       −0.58     −0.57     −0.64         0.51           0.51       −0.53         0.58       −0.58        −0.53        −0.57         0.74       0.64       −0.51  −0.54    −0.57	0.64
33	♀ 0.67	B 0.65	−0.58        −0.58         0.53       0.54       −0.50         0.56       0.54       −0.65         0.56           0.56       0.50        0.60         −0.54         0.54           0.51           0.57           0.61           0.50      0.54       0.50	0.52
34	5 0.55       B 0.63	−0.70         0.54       0.52       0.55       0.53        0.53           0.53        0.59           0.51         −0.65        −0.61         0.54           0.51           0.55           0.51           0.51           0.54        0.56     0.51       0.51	0.51
35	↑ 0.61	0.66          0.53       0.68        0.53          0.55       0.52       0.51        0.68           0.58         −0.64       −0.59         0.50           0.53           0.53          0.53      −0.52         0.53        0.53          0.54        0.53       0.62
36	♀ 0.55	B 0.57	−0.66         0.52       0.57       0.51      −0.51         0.50           0.53           0.60      −0.56      0.51        0.54           0.52         −0.58         0.51           0.53           0.56           0.55        0.50       0.52       0.51	0.68
37	♂ 0.68	B 0.67	0.52      −0.52         0.66           0.57         −0.56       −0.58       −0.50       −0.56     −0.56         0.63      −0.52       −0.51       −0.51         0.55         −0.56      0.59       0.60           0.52     −0.56       −0.54	0.64
38	↑ 0.62      W 0.58	−0.64         0.50          0.64       0.53       0.53        0.55          0.52       0.57      −0.51         0.50        0.54           0.52          0.57        0.52          0.57       0.56       0.51        0.51     0.51       0.50	0.67
39	♂ 0.63      ↑ 0.66      W 0.77	0.50       0.60         −0.62       −0.55         0.64         −0.54       −0.50         0.70        −0.53     −0.58         0.57        −0.59      0.55       0.53         −0.50       −0.68     −0.53         0.53          0.63          0.54    −0.60
40	♀ 0.58	B 0.68	0.53       0.53       0.52       0.62       0.56       0.58       0.60       0.69       0.56          0.57       0.54       0.62       0.51       0.51          0.54       0.61       0.57       0.53          0.57       0.52	0.58
41	♀ 0.56       ↓ 0.74       B 0.79	−0.56        −0.57         0.51        0.54         −0.56     −0.58     −0.59      −0.65          0.52           0.65       −0.51          0.58          −0.59        −0.52       −0.51       0.70       −0.58        −0.52  −0.59         0.56	0.52
42	♂ 0.62      ↑ 0.55      W 0.74	0.67        0.62         −0.58       −0.56         0.58       −0.55       −0.54         0.68      −0.59     −0.56         0.56      −0.60         0.63      −0.52       −0.56       −0.66     −0.54       −0.51       −0.67       −0.53   −0.57
43	5 0.56     W 0.68	−0.56         0.52      −0.53       −0.55       −0.52       −0.51         0.52        0.69         −0.52       −0.67       −0.59       −0.56         0.59           0.51           0.59         −0.59     −0.55         0.50           0.60           0.54   −0.64

44	↑ 0.80	0.71          0.55      −0.64         0.51       0.50        0.59           0.54          0.69       0.53        0.51          0.61       0.50       0.65        0.57           0.61         −0.56      0.61           0.60          0.63        0.52    −0.57
45	♀ 0.63	B 0.76	−0.56        −0.54         0.56          0.57      −0.58         0.54        0.54         −0.66         0.60          0.61      −0.60         0.64          0.53        0.51           0.51          0.64       0.50        0.53         0.58       0.53	0.55
46	♀ 0.66       5 0.57       B 0.55	−0.55         0.60           0.62          0.55      −0.67         0.52          0.54      −0.70         0.59          0.53      −0.57         0.61       0.50        0.53           0.54          0.58       0.52        0.51         0.51       0.54	0.53
47	♂ 0.58      ↑ 0.63      W 0.76	0.69           0.52          −0.53       −0.54       −0.61       −0.55         0.61           0.61         −0.54       −0.55       −0.54       −0.56         0.60           0.51         −0.51       −0.65      0.63           0.52           0.58          −0.51      −0.50
48	♀ 0.62      ↓ 0.82      B 0.56	0.61      −0.50      0.67          −0.55       −0.55       −0.53       −0.54       −0.72     −0.56       −0.57       −0.54      0.54      −0.58       −0.56       −0.56         0.54         −0.59     −0.52       −0.60       −0.51	0.65
49	♂ 0.59      ↑ 0.67      W 0.80	0.71          0.52         −0.58       −0.55      0.56      −0.54       −0.56        0.57      −0.57     −0.51         0.55         −0.60        0.55       0.55         −0.53       −0.72     −0.64         0.56          0.53         −0.54     −0.63
50	♂ 0.59      ↑ 0.66      W 0.69	−0.51         0.60         −0.55       −0.55        0.58      −0.53       −0.52        0.51      −0.54      0.63       0.51      −0.57      0.54       0.51         −0.53       −0.64     −0.53       −0.53         0.60         −0.56   −0.57
51	♀ 0.70       ↓ 0.66       B 0.72	−0.51        −0.52         0.55           0.58       0.63        0.54           0.51       −0.58         0.58           0.66       −0.50         0.63         −0.53     −0.52        −0.53         0.67       0.65        0.56   −0.66      0.51	0.61
52	♂ 0.56      5 0.55	0.53          0.56       0.67          0.52          0.60          0.54          0.50          0.59         −0.52       −0.69       −0.51       −0.50         0.57          0.52          0.56        −0.54      0.59       0.53          0.52       0.54      0.61
53	5 0.58     W 0.72	0.61       0.50      −0.55       −0.52       −0.53       −0.56       −0.58       −0.68     −0.52       −0.53       −0.55       −0.50         0.59           0.56         −0.52       −0.59      0.51      −0.55       −0.53         0.51     −0.53

54	♂ 0.65      ↑ 0.67      W 0.75	0.64       0.52      −0.55    −0.52      0.51      −0.52        0.59       0.60      −0.58     −0.54       −0.58       −0.60        0.55          0.52        −0.51      −0.69     −0.62        0.53       0.54        −0.52      −0.57
55	↓ 0.65       B 0.73	0.58       −0.50         0.52           0.58       0.66        0.53           0.51       −0.59         0.52           0.65       −0.53         0.59         −0.58     −0.52        −0.57         0.64       0.51       −0.52   −0.67     −0.56	0.64

56	♂ 0.65      ↑ 0.59      W 0.83	0.51   0.54         −0.65        −0.61        −0.64        −0.61        −0.51         0.68         −0.57     −0.55        −0.59       −0.63     −0.58     −0.50        −0.58       −0.71     −0.51        −0.50        −0.53        −0.52        −0.57
57	♂ 0.63      ↑ 0.71      W 0.73	0.71          0.54         −0.54       −0.51        0.60      −0.51        0.57       0.55      −0.51       −0.52        0.53      −0.54         0.52          0.52          0.57      −0.68      0.60          0.50          0.56         −0.55     −0.55
58	B 0.62	0.51        0.52          0.53       0.57        0.67           0.55          0.57        0.68           0.53          0.51        0.50           0.59          0.55        0.53           0.55          0.57       0.55        0.53   −0.62        0.51	0.61

59	♂ 0.64       ↓ 0.59       B 0.71	0.52       −0.53         0.52        0.60         −0.57        −0.52        −0.50        −0.58         0.54           0.67       −0.53         0.51       −0.56         0.52         −0.58         0.66        0.63       −0.57   −0.56      0.51	0.58
60	♂ 0.62     ↓ 0.67	0.57         −0.53       −0.62     −0.55       −0.53       −0.55       −0.55       −0.60       −0.56     −0.57       −0.53       −0.55        −0.52       −0.54       −0.58       −0.52       −0.55       −0.54       −0.58       −0.54    0.62
61	♂ 0.55      ↑ 0.63      W 0.55	−0.57        0.54      −0.67        0.57       0.54       0.59          0.55          0.53       0.54         −0.60         0.63         −0.55         0.51          0.53         −0.53       −0.58      0.58          0.55          0.55       0.51    −0.64
62	♂ 0.55       5 0.56       B 0.59	0.54       0.52       0.52       0.55       0.70       0.53       0.54      −0.70         0.54          0.55      −0.59         0.54      −0.55         0.53         −0.52         0.57       0.52        0.51         0.53      −0.56	0.54
63	♀ 0.57       ↓ 0.60       B 0.72	0.68      −0.51         0.62       0.56      −0.66         0.55       0.50      −0.75      0.56          0.54       0.57       0.61      −0.61     −0.52         0.50       0.70       0.51      −0.56      −0.58      0.51	0.59
64	♂ 0.67      ↑ 0.64      W 0.70	0.59           0.52         −0.53       −0.54         0.57      −0.55     −0.55      0.65         −0.51       −0.50       −0.59       −0.54         0.54           0.53         −0.51       −0.62     −0.62         0.51           0.59           0.56    −0.56
65	5 0.55       B 0.68	0.50          0.53       0.54       0.61       0.50        0.54          0.54      −0.58      0.55          0.51      −0.57         0.57           0.50           0.53           0.53          0.60       0.61        0.56      0.51       0.55	0.60

66	♀ 0.69       ↑ 0.64       B 0.56	−0.58         0.60           0.59           0.54           0.63           0.58           0.57           0.55       0.54       0.51       0.54       0.57       0.57        0.53           0.59           0.56         −0.53         0.54           0.53           0.52       −0.57
67	5 0.55       B 0.69	0.52       0.55       0.52       0.57        0.67           0.53          0.54        0.58           0.54          0.50      −0.61         0.56           0.51           0.53           0.53           0.59          0.58        0.55      −0.66         0.51	0.59

68	♂ 0.61       ↓ 0.63       B 0.79	0.53       −0.52         0.57       0.50       −0.53       −0.52      0.57       −0.58         0.56           0.54       −0.51         0.54       −0.57         0.55         −0.56         0.71       0.63       −0.52   −0.63      0.56	0.61
69	♀ 0.55	B 0.54	0.66          0.51      −0.66         0.51       0.50        0.55          0.56        0.71           0.52           0.61         −0.61         0.54           0.52           0.53           0.52         −0.54      0.50        0.51       0.51       0.56	0.53
70	♀ 0.55       ↓ 0.67       B 0.76	0.53       −0.54         0.51        0.54       −0.55         0.61         −0.58     −0.63         0.50           0.64      −0.56       0.58          −0.51     −0.52        −0.51         0.66        0.58       −0.54    −0.64       −0.57	0.63
71	B 0.62	0.70          0.53       0.53       0.57        0.64           0.56          0.52       0.61        0.51          0.50      −0.55         0.52         −0.51         0.52         −0.53         0.55          0.56        0.50         0.52       0.52	0.58

72	↑ 0.58	−0.52      0.55       0.68          0.52       0.57       0.51          0.51       0.56      −0.55       −0.61       −0.59       −0.57       −0.60         0.54          0.54        −0.52      0.55       0.54          0.54       0.51   0.67

73	♀ 0.73       ↓ 0.74       B 0.60	−0.52     −0.51       0.53           0.54       0.53       0.58       0.52       −0.67         0.55         −0.60       −0.50       0.58         −0.61        −0.52        −0.51         0.62       0.56       −0.53    −0.52      0.57	0.55
74	♀ 0.54       ↑ 0.78      W 0.67	−0.61         0.58      −0.55         0.60       0.51       0.54        0.52          0.70       0.52       0.53       0.56      −0.52      0.61        0.55          0.58      −0.65      0.60           0.55          0.60        0.52     −0.57
75	↓ 0.70      B 0.64	0.62      −0.52         0.54        0.56         −0.51       −0.52       −0.52       −0.64       −0.54         0.67      −0.51      0.54         −0.62       −0.54       −0.56         0.58          0.58      −0.56     −0.60       −0.52	0.54

76	♂ 0.58       ↓ 0.68       B 0.75	0.55       −0.54         0.57        0.52       −0.53        −0.51       −0.54     −0.63         0.57           0.50       −0.55         0.55      −0.58     −0.55        −0.57         0.72        0.60       −0.50  −0.55    −0.57	0.65
77	♂ 0.61      ↑ 0.59      W 0.74	−0.68         0.60         −0.56       −0.54       −0.66       −0.54       −0.52         0.69         −0.56       −0.52         0.60         −0.59        0.54      −0.57      −0.53    −0.63     −0.50       −0.50         0.66         −0.57      −0.63
78	↓ 0.71       B 0.80	0.71      −0.56      0.51        0.54         −0.60     −0.58     −0.53        −0.61          0.52           0.65      −0.60       0.56          −0.57        −0.52        −0.53         0.69       −0.56        −0.50     −0.56     −0.54	0.55

79	B 0.58	0.69           0.60           0.65       0.54       0.60       0.52       0.52       −0.66       −0.52     −0.51        −0.54         0.55         −0.59         0.52           0.51         −0.54         0.52        0.54         0.66       0.58	0.57

80	♀ 0.63       ↓ 0.64       B 0.64	−0.62     −0.51      0.53       0.55      −0.68      0.54       0.51      −0.57         0.56          0.53      −0.50         0.61      −0.55     −0.51         0.57          0.65       0.51      −0.51       −0.59      0.52	0.61
81	↑ 0.55     W 0.78	−0.69         0.52      −0.55       −0.52         0.60      −0.54       −0.60      0.69         −0.57     −0.55       −0.57       −0.57       −0.53       −0.55       −0.52       −0.68     −0.64      0.52           0.59         −0.52      −0.57

82	♀ 0.62	W 0.62	0.70       −0.55        −0.56        −0.52        −0.64        −0.53         0.62           0.70        0.51           0.55        0.56        0.51           0.60       −0.57         0.55          −0.51        −0.50         0.52         −0.67      0.50    −0.60
83	↓ 0.65       B 0.79	0.57      −0.50      0.57       0.58      −0.65     −0.52         0.52       −0.55         0.53           0.55      −0.51      0.60      −0.54     −0.58        −0.54         0.73       0.54       −0.55    −0.50      0.55	0.65

84	♀ 0.55      ↑ 0.66      W 0.83	−0.58         0.51         −0.59       −0.57         0.60         −0.50       −0.53         0.54      −0.54     −0.51         0.51      −0.57      0.52      −0.51       −0.50       −0.71     −0.56       −0.51         0.57         −0.56     −0.63
85	↓ 0.65       B 0.86	−0.64     −0.54         0.57       0.59       −0.53         0.51       0.51       −0.58         0.56           0.53      −0.61      0.63       −0.55         0.57         −0.51         0.74       0.53       −0.56   −0.52      0.53	0.63

86	↑ 0.60     W 0.82	0.50       0.53      −0.50       −0.56         0.66         −0.52        0.61      −0.57       −0.53     −0.61         0.56         −0.55        0.52      −0.58       −0.61     −0.64      0.64         −0.52         0.59         −0.52    −0.52

87	♀ 0.66	B 0.60	−0.60         0.51        0.57          0.58       0.50        0.55          0.58        0.69           0.58           0.61           0.58           0.60           0.52           0.54           0.53           0.54          0.51        0.54     0.54       0.51	0.51
88	♀ 0.68       ↓ 0.54       B 0.56	−0.60     −0.60      0.67           0.51      −0.54         0.50           0.52      −0.58         0.55           0.53       0.52        0.58         −0.60      0.55           0.53       0.57      −0.55        −0.58       −0.57         0.51	0.55
89	5 0.56	0.53      −0.51       −0.53       −0.59       −0.51       −0.51       −0.50       −0.54         0.56          0.53       0.61        0.56         −0.51       −0.57       −0.53         0.51         −0.51       −0.52       −0.66         0.55       0.66

90	♂ 0.56	W 0.79	−0.54        −0.56        −0.56        −0.56        −0.55        −0.56        −0.56         0.70      −0.58     −0.61       −0.56     −0.59     −0.51        −0.52        −0.56       −0.71     −0.51        −0.52         0.57         −0.53  −0.63
91	↓ 0.67       B 0.85	0.69       −0.55         0.56       0.54       −0.52         0.52       0.51       −0.62         0.55           0.53       −0.52         0.61       −0.56        −0.56       −0.54      0.74       0.64 −0.55       −0.52      0.51	0.63

92	♀ 0.56       ↑ 0.68      W 0.61	−0.65         0.55      −0.56         0.57        0.58           0.50           0.51          0.57        0.53          0.54       0.54        0.51          0.58        0.55          0.55      −0.57         0.60           0.53           0.54           0.52       −0.51
93	♂ 0.58      ↑ 0.64      W 0.63	−0.62         0.58          0.61      −0.59      0.57      −0.54     −0.52        0.52      −0.55     −0.58        0.52      −0.50         0.54          0.58         −0.58      −0.63     −0.62         0.59          0.51      −0.50  −0.55
94	5 0.58       B 0.72	0.70          0.53        0.51          0.58       0.56        0.52          0.57      −0.68         0.58          0.54      −0.57         0.59          0.53        0.54          0.53        0.61          0.59        0.55       0.52       0.55	0.60

95	♀ 0.58       5 0.58       B 0.73	0.57      −0.61      0.56       0.58       0.65       0.52       0.54      −0.51         0.59          0.55      −0.61      0.62      −0.52         0.52          0.51       0.67       0.53       0.50        0.62       0.51	0.67
96	↑ 0.57	−0.64      0.56       0.66          0.53       0.51       0.51          0.52          0.57       0.54          0.54          0.53          0.50        −0.60        0.54          0.51        −0.60        0.55       0.55          0.53       0.51    0.54

97	♀ 0.55       ↓ 0.75       B 0.78	−0.62     −0.59         0.52        0.51          −0.51        −0.52       −0.59     −0.63          0.53           0.52      −0.60      0.59       −0.58        −0.51        −0.54         0.70       −0.52        −0.52      −0.57      0.55	0.60
98	♂ 0.57      ↓ 0.58      W 0.69	0.68           0.58          −0.53        −0.54        −0.58        −0.56        −0.57        −0.53        −0.57     −0.59        −0.58        −0.50        −0.53        −0.54        −0.59        −0.56         0.60         −0.55        −0.55        −0.51   −0.58
99	♂ 0.65      ↓ 0.57	0.55      −0.55     −0.59       −0.51       −0.55       −0.59       −0.50     −0.61       −0.53         0.51      −0.55       −0.51       −0.55     −0.50       −0.62         0.51          0.54      −0.51       −0.65     −0.56       0.55
100	♀ 0.57       ↑ 0.73      W 0.71	−0.51         0.53         −0.50        −0.52         0.62           0.57           0.54           0.62      −0.51       0.56           0.52      −0.52      0.59        0.57           0.54      −0.64     −0.52         0.50           0.60        0.57       −0.57
101	♀  0.62        ↑  0.58     W 0.64	0.66        0.51           0.63        0.50        0.61           0.53           0.60        0.67        0.54           0.53        0.51        0.55           0.53        0.54           0.52        0.56        0.51        0.51   0.53       0.51	0.54
102	↑ 0.62     W 0.90	0.64       0.51         −0.60       −0.59         0.61        −0.53     −0.61      0.65        −0.56     −0.52        0.54      −0.59      0.58      −0.52       −0.51     −0.72     −0.56       −0.51         0.62         −0.51   −0.64

103	♀ 0.66       ↓ 0.58       B 0.66	−0.55       −0.60      0.57        0.51         −0.51         0.53        0.52         −0.70         0.55           0.55        0.60           0.58         −0.61        −0.51         0.53        0.63        0.58         −0.50      0.57       0.51	0.50
104	♀ 0.70	B 0.63	−0.53         0.54       0.56       0.57        0.66          0.57       0.62      −0.68         0.57          0.53       0.52        0.60           0.62           0.52           0.57          0.57       0.59        0.55     0.51       0.51	0.57
105	♂ 0.59      5 0.60     W 0.67	0.53       0.52         −0.58       −0.52        0.56      −0.51         0.61        −0.55     −0.52       −0.59       −0.55       −0.52         0.56           0.55         −0.51       −0.54        0.58       0.51          0.57          0.50	0.59
106	♂ 0.55	W 0.68	0.70          0.50      −0.50       −0.52        0.57      −0.52       −0.53       −0.66       −0.53       −0.55       −0.59       −0.54        0.51      −0.52       −0.51      −0.58     −0.52       −0.57       −0.61       −0.54       −0.52
107	♀ 0.61       ↑ 0.58       B 0.61	−0.73         0.56      −0.67         0.53        0.67           0.54           0.55           0.59           0.53          0.57        0.59           0.56          0.55        0.54          0.52        0.52           0.59           0.53      0.58       0.52	0.66
108	↑ 0.55     W 0.65	0.60       −0.59        −0.51        −0.51        −0.52        −0.54        −0.52         0.64         −0.51        −0.53        −0.53        −0.52        −0.54        −0.50        −0.53        −0.57        −0.51        −0.51        −0.57         0.52  −0.50

109	♂ 0.67	W 0.56	0.52      −0.61       −0.52       −0.51        0.64      −0.55       −0.51       −0.52       −0.55       −0.67     −0.54       −0.53       −0.51       −0.57       −0.54         0.55         −0.51       −0.51       −0.55       −0.54	0.61
110	♂ 0.59	W 0.74	−0.67         0.53       −0.57        −0.55        −0.54        −0.56        −0.53        −0.67       −0.59     −0.51        −0.58       −0.60     −0.55        −0.55        −0.54       −0.65     −0.51        −0.52        −0.61        −0.51   −0.51
111	♀ 0.60       ↑ 0.67      W 0.67	0.64           0.53         −0.52        −0.50        −0.55         0.58           0.57           0.54        0.52           0.59           0.52           0.56        0.55        0.51           0.51         −0.61       0.55           0.51           0.53           0.53    −0.54
112	♀ 0.54	W 0.60	0.51        0.62          −0.58         0.53       −0.61        −0.51         0.54       −0.54          0.55         −0.64     −0.53          0.55         −0.60     −0.54        −0.51          0.53           0.54       −0.55   −0.64      0.58	0.65
113	♀ 0.74       ↑ 0.56       B 0.59	−0.54       −0.55         0.56        0.50         −0.59         0.58       0.56       0.55        0.59          0.52       0.52        0.62          0.56      −0.54         0.60       0.65        0.60           0.56      0.54       0.52	0.62
114	↓ 0.54       B 0.81	−0.72        −0.55         0.51       0.56      −0.57         0.51          0.50      −0.51         0.57          0.51       0.54       0.62      −0.58         0.51           0.52          0.71       0.55        0.55         −0.63      0.51	0.58

115	♂ 0.55	W 0.68	0.64       0.57      −0.52       −0.56      −0.62     −0.56       −0.51       −0.61      −0.56     −0.53       −0.51       −0.53       −0.54       −0.55       −0.55       −0.58    −0.60     −0.53       −0.56       −0.51       −0.58
116	♀ 0.56      5 0.56      W 0.71	−0.56         0.52         −0.56       −0.51      −0.55     −0.52       −0.52      −0.70      0.53         −0.53       −0.57       −0.50         0.55         −0.51       −0.51       −0.59        −0.55       −0.51        0.57       0.52      −0.55
117	↑ 0.58	0.59          0.57      −0.71        0.52       0.54       0.51          0.53          0.63          0.50        −0.59      0.51          0.54       0.58       0.53          0.53         −0.52        0.54       0.55          0.52          0.54   −0.62

118	♂ 0.59      ↑ 0.77      W 0.61	0.68          0.59        −0.58        0.57          0.52       0.51          0.54          0.56      −0.54      0.62          0.52      −0.51      0.58          0.53          0.52        −0.63      0.56          0.55          0.57       0.51    −0.52
119	♀ 0.64       ↑ 0.63       B 0.56	−0.58        0.51       0.59       0.52       0.60          0.57       0.57       0.58       0.55       0.56       0.55       0.55          0.61       0.52          0.63       0.59          0.57           0.51          0.56       0.52	0.53
120	♂ 0.60       ↑ 0.59       B 0.63	0.61       0.50       0.53       0.56        0.67           0.52          0.54      −0.70       −0.56      0.52      −0.50         0.50           0.50           0.55           0.50           0.54          0.59        0.55       0.57       0.52	0.56
121	♂ 0.59      ↓ 0.55      W 0.66	0.52       −0.59        −0.55        −0.57        −0.52        −0.57        −0.50       −0.59     −0.57        −0.52        −0.53        −0.58        −0.52        −0.52        −0.57       −0.60      −0.54        −0.52        −0.57        −0.55  −0.50
122	↑ 0.67      W 0.75	−0.50         0.55       −0.51        −0.53         0.53       −0.56         0.58       0.59       −0.53        −0.57         0.52       −0.55         0.56        0.50           0.52      −0.65       0.61           0.51           0.53           0.57    −0.58

123	♂ 0.59      ↑ 0.60      W 0.66	0.58       0.52      −0.51       −0.51       −0.56       −0.51       −0.51         0.61         −0.53       −0.56       −0.56       −0.56       −0.55         0.56         −0.51       −0.63      0.57        0.54           0.60         −0.51     −0.65
124	♀ 0.61       ↓ 0.55       B 0.70	−0.61         0.52       0.56       0.61       0.59       0.55       0.60      −0.73      0.54          0.52       0.51        0.60         −0.58         0.52           0.54           0.59          0.59        0.53      0.54       0.52	0.61
125	5 0.56     W 0.59	−0.74        0.56       0.67          0.51       0.52       0.58          0.53      −0.70       −0.51       −0.50     −0.59       −0.57       −0.57      0.51         −0.57       −0.54      0.56       0.52          0.56         −0.57   −0.67

126	♀ 0.62	0.66      −0.59       −0.68        0.51       0.52      −0.59         0.50         −0.55         0.53          0.62      −0.54         0.57          0.59      −0.56       −0.51         0.52          0.56       0.58          0.71          0.52    0.52
127	↓ 0.64     W 0.55	−0.52        −0.51        −0.53        −0.51        −0.54        −0.55        −0.56        −0.57        −0.53        −0.60        −0.50        −0.55        −0.55        −0.56        −0.54       −0.56      −0.53        −0.54  −0.59       −0.54	0.59

128	♀ 0.71       ↓ 0.73       B 0.70	0.67       −0.55         0.51        0.51          −0.53         0.55        0.54       −0.53          0.55           0.62      −0.56       0.59         −0.55     −0.51       −0.53      0.64       −0.55        −0.52   −0.54       −0.55	0.50
129	♀ 0.63       ↓ 0.63       B 0.68	−0.55        −0.50         0.56           0.60       0.63        0.54           0.52      −0.61      0.53           0.56      −0.59      0.59         −0.56     −0.54       −0.57      0.61           0.58      −0.51       0.67       0.57	0.54
130	♀ 0.57       ↑ 0.70       B 0.59	−0.67        0.52       0.59       0.60       0.56          0.62       0.64       0.64       0.57          0.56       0.55          0.60          0.60       0.55          0.63       0.61       0.51       0.58          0.61      0.56	0.51
131	♀ 0.56       ↓ 0.69       B 0.83	−0.58        −0.54         0.52        0.52        0.59        0.57         −0.58     −0.62          0.55           0.59      −0.55       0.62          −0.54        −0.52        −0.53         0.70        0.62          −0.53   −0.56       −0.56	0.55
132	↑ 0.58     W 0.79	−0.58         0.51         −0.59        −0.54        −0.62        −0.54        −0.59         0.65         −0.53        −0.51         0.59         −0.54         0.62         −0.54        −0.50       −0.66     −0.52         0.54           0.55         −0.56     −0.56

133	♀ 0.71       5 0.57	−0.54         0.53        0.55          0.57       0.53       0.54       0.60      −0.51         0.57          0.60      −0.60         0.60         −0.60     −0.56         0.50          0.54       0.58        0.50          0.56       0.52      0.54
134	♂ 0.56       5 0.59       B 0.58	0.70       0.54        0.60          0.56       0.52        0.58           0.52           0.59           0.52          0.51      −0.52         0.52         −0.58         0.54         −0.54         0.51          0.57        0.57         0.59       0.51	0.54
135	♀ 0.75       ↓ 0.63       B 0.65	−0.56     −0.54         0.54        0.51         −0.53         0.53       0.54       −0.54         0.58           0.52       0.59        0.60         −0.53     −0.51         0.55       0.66       −0.54        −0.51    −0.67      0.57	0.50
136	↑ 0.57     W 0.73	−0.64     −0.54     −0.57       −0.53     −0.54       −0.53       −0.51         0.63      −0.51       −0.51         0.57      −0.57      0.58         −0.51       −0.52       −0.67     −0.56       −0.51       −0.56       −0.56       −0.64

137	♀ 0.57      ↓ 0.56      W 0.58	0.62       0.55         −0.59         0.60          −0.51       −0.51       −0.52       −0.64     −0.51      0.64       0.54      −0.51     −0.50       −0.57         0.57          −0.54       −0.51       −0.52       −0.65      0.57      −0.58
138	↓ 0.75      B 0.55	−0.58       −0.53         0.62           0.59         −0.63     −0.56     −0.52       −0.56       −0.53         0.58         −0.57      0.55         −0.60     −0.56       −0.54         0.56          0.61      −0.53     −0.58       −0.52	0.65

139	♂ 0.57	W 0.71	0.61       −0.51        −0.56        −0.55        −0.64        −0.60        −0.51        −0.51        −0.55        −0.56        −0.50        −0.55        −0.50        −0.50        −0.59       −0.61      −0.57        −0.56        −0.62        −0.52  −0.50
140	♀ 0.59      ↓ 0.61      W 0.76	0.70   0.54         −0.53        −0.58        −0.52        −0.54        −0.53        −0.53       −0.54     −0.54        −0.53       −0.55      −0.57        −0.56        −0.55       −0.63     −0.51        −0.56        −0.54        −0.52        −0.57
141	♀ 0.55      5 0.55      W 0.66	−0.55         0.57         −0.54       −0.53       −0.60       −0.59       −0.54       −0.65         0.55          0.52      −0.57         0.58          0.52        0.53           0.59         −0.54         0.58      −0.53         0.55        0.50    −0.57
142	♂ 0.65      ↑ 0.61      W 0.85	0.53        0.50         −0.61        −0.58        −0.61        −0.59        −0.51        −0.58       −0.59     −0.53        −0.59        −0.62        −0.53        −0.54        −0.56       −0.71     −0.58        −0.51         0.63         −0.53   −0.58
143	♂ 0.63	W 0.65	0.62      −0.52       −0.57       −0.58      0.62       −0.58       −0.55       −0.57       −0.53       −0.57       −0.58        −0.54       −0.54       −0.56       −0.56       −0.58       −0.59       −0.54       −0.52       −0.56       −0.56
144	5 0.55	−0.59        0.52      −0.69         0.50         −0.56       −0.54         0.51          0.67          0.53          0.54      −0.58         0.50         −0.58         0.52          0.51      −0.52        0.51       0.55         −0.68       −0.54      0.55

145	♂ 0.56      5 0.61     W 0.62	0.67        0.59         −0.52       −0.53         0.58      −0.56       −0.51         0.67         −0.54       −0.57         0.58         −0.51       −0.54         0.55         −0.52       −0.59       −0.54         0.57           0.61           0.56       −0.54
146	♀ 0.67      ↓ 0.61      B 0.57	0.50      −0.51         0.70          0.58        −0.62      0.56          0.50      −0.58         0.54         −0.54       −0.54         0.60         −0.54       −0.55         0.62          0.57          0.61      −0.50     −0.55        0.53	0.52
147	↓ 0.63       B 0.79	0.53      −0.63      0.59       0.60       0.51        0.59          0.51      −0.55         0.55          0.57      −0.53         0.59      −0.53         0.54         −0.51      0.67       0.56        0.50      −0.53     0.52	0.67

148	♀ 0.60       ↑ 0.74	−0.70         0.52      −0.67         0.50       0.53        0.56           0.50          0.60        0.55          0.50       0.53        0.54          0.61        0.55           0.57         −0.52         0.59           0.55           0.53           0.53     −0.63
149	♀ 0.65 ↓ 0.67 W 0.74 −0.54 −0.55 −0.52 −0.55 −0.58 −0.52 −0.52 −0.54 −0.53 −0.57 −0.57 −0.53 −0.55 −0.56 −0.56 −0.61 −0.60 −0.55 −0.52 −0.51 −0.57
150	♀ 0.64      ↓ 0.67      B 0.57	0.69      −0.55         0.63           0.55         −0.52         0.55         −0.57       −0.53         0.51         −0.52         0.55       0.51      −0.53     −0.54       −0.55         0.53         −0.57       −0.54       −0.54       −0.50      −0.55
151	♂ 0.56      ↑ 0.59      W 0.60	−0.57      0.51         −0.61       −0.62       −0.60       −0.56         0.51           0.62         −0.56       −0.61       −0.58       −0.56         0.59           0.53           0.55         −0.57      0.59           0.51          0.57        0.50     −0.66
152	♀ 0.59       5 0.57      W 0.57	0.56        0.55         −0.55         0.52       0.57        0.55          0.51      −0.63         0.52          0.57        0.59           0.52          0.51        0.58          0.50      −0.54         0.55        0.52      0.60       0.52	0.68
153	♀ 0.56       ↓ 0.63       B 0.79	0.71      −0.62      0.56       0.61       0.51       0.52       0.51      −0.55         0.54          0.61      −0.52         0.62      −0.57      0.55         −0.56         0.66       0.54        0.51       −0.50      0.53	0.66
154	♀ 0.63      ↑ 0.72	−0.65         0.53          0.64          0.52       0.61          0.58          0.51          0.58       0.55          0.56          0.55       0.54       0.60       0.52          0.58         −0.51       −0.55         0.54          0.56          0.54    −0.54
155	↓ 0.66       B 0.80	0.52       −0.53         0.54       0.57       −0.56         0.57       0.59       −0.63         0.53           0.55       −0.51         0.59       −0.56        −0.55        −0.54         0.74       0.52 −0.53       −0.52      0.57	0.63

156	↑ 0.55     W 0.66	−0.62        0.54      −0.56        0.59       0.59         −0.50        0.51      −0.65       −0.54         0.66         −0.50       −0.51         0.60          0.54          0.59         −0.55        0.51      −0.53         0.54        −0.50	0.62

157	♀ 0.58	W 0.70	0.54       0.63         −0.51      −0.54      0.65      −0.52       −0.51        −0.54       −0.57       −0.51       −0.51         0.56         −0.54       −0.54       −0.51       −0.56        0.54      −0.50       −0.52       −0.55       −0.53
158	↓ 0.59       B 0.64	0.72           0.53        0.64           0.55       0.66       0.58       0.55       −0.51         0.53         −0.66        −0.52         0.55       −0.50        −0.57        −0.52         0.59       0.54        0.50     −0.56         0.51	0.53

159	♀ 0.66       ↓ 0.58       B 0.63	0.75      −0.59      0.54       0.56       0.70          0.50       0.51      −0.56         0.56          0.52      −0.58      0.60      −0.56      0.51          0.54       0.59       0.51      −0.55      0.52      0.52	0.55
160	♀ 0.63       ↑ 0.71      W 0.68	−0.58         0.54      −0.51         0.60      −0.62         0.51           0.52          0.60        0.53          0.52        0.51           0.54          0.60        0.51          0.58      −0.63      0.62           0.53          0.55        0.53        −0.50
161	♂ 0.62      ↑ 0.62      W 0.79	0.52        0.59         −0.61        −0.57        −0.54        −0.58        −0.52        −0.60        −0.56        −0.56        −0.57        −0.58         0.58           0.50         −0.54       −0.66     −0.52         0.53    0.66         −0.54       −0.51
162	↓ 0.64       B 0.82	0.52       −0.54         0.59       0.57      −0.58      0.57       0.52       −0.57         0.53           0.50       −0.51         0.59       −0.55        −0.58        −0.54         0.72       0.52       −0.56  −0.54     −0.57	0.67

163	♀ 0.55	0.60       0.54       0.70          0.54      −0.68        0.57       0.59         −0.55       −0.53       −0.66       −0.62         0.53         −0.51       −0.56       −0.54         0.53          0.50      −0.55       −0.52       −0.53        0.68
164	♀ 0.61       ↓ 0.56       B 0.68	−0.66       −0.54         0.55       0.54      −0.51         0.52       0.55       0.70        0.58          0.57      −0.55      0.61       0.54        0.54          0.50       0.67      −0.60      0.50      −0.68      0.52	0.57
165	♂ 0.55      ↓ 0.74      B 0.63	−0.51        −0.56         0.63           0.54         −0.51        −0.50        −0.57        −0.61       −0.53     −0.51        −0.55        −0.52        −0.60        −0.53        −0.61         0.51          −0.56        −0.55    −0.60       −0.51	0.67
166	↓ 0.73      B 0.77	−0.69     −0.53        0.53          0.56      −0.52       −0.51       −0.53       −0.63        0.52          0.60      −0.54        0.58        −0.57       −0.53       −0.53        0.64          0.55      −0.52       −0.57       −0.52	0.62

167	↓ 0.69       B 0.81	−0.60       −0.58      0.52       0.57       −0.51         0.58           0.52       −0.63         0.55           0.58      −0.58      0.61       −0.57        −0.55       −0.55      0.73       0.53       −0.55    −0.54      0.56	0.66

168	♀ 0.63       ↓ 0.71       B 0.75	0.72       −0.54         0.53       0.58      −0.59      0.53       0.51      −0.64         0.56          0.61      −0.50      0.62      −0.56     −0.53       −0.57      0.69       0.55      −0.51       −0.55     −0.56	0.65
169	↓ 0.61       B 0.77	0.52      −0.52         0.54           0.58       0.65        0.60           0.51      −0.56         0.54           0.55      −0.50         0.61      −0.54        −0.55        −0.52         0.71       0.53        0.55       −0.50      0.52	0.65

170	↓ 0.59      B 0.65	0.54      −0.55        0.52       0.51         −0.53       −0.53        0.59      −0.64         0.57          0.55      −0.56         0.55         −0.57       −0.57       −0.53        0.65       0.53      −0.52      −0.56       −0.57	0.63

171	♀ 0.63       ↑ 0.60       B 0.73	−0.66       −0.59         0.54       0.57        0.59           0.56           0.52          0.54        0.60          0.52       0.52        0.62          0.55        0.55           0.55          0.61      −0.62         0.55        0.52          0.56	0.53
172	♀ 0.65      ↓ 0.67      B 0.72	−0.71     −0.57         0.57          0.51         −0.57       −0.53       −0.57       −0.52         0.58          0.55      −0.55      0.63      −0.53       −0.53       −0.51        0.67      −0.56       −0.52     −0.52     0.50	0.60
173	♂  0.56      ↑ 0.65	−0.60      0.59      −0.67      0.52       0.54        0.50          0.55       0.58       0.57        0.66           0.52         −0.57         0.55           0.57           0.53         −0.56      0.53        0.57          0.63        0.51   −0.66
174	♀ 0.59	B 0.81	−0.57       −0.51         0.57       0.56      −0.54         0.53       0.53       0.50        0.57          0.56       0.54        0.61          0.53        0.51           0.50          0.63       0.58        0.51   −0.60     0.53	0.51
175	♂ 0.68      ↑ 0.66      W 0.76	0.62       0.50        −0.53       −0.56        0.51      −0.52    −0.59      0.58      −0.59     −0.57        0.55      −0.61      0.52       0.53        −0.54      −0.69    −0.63      0.54          0.54        −0.51     −0.58
176	♀ 0.69       ↓ 0.63       B 0.66	−0.63      0.59       0.51          0.60       0.60       0.52       0.59      −0.53         0.58          0.55      −0.61      0.63      −0.52      0.55           0.50          0.66       0.60       0.53        0.51      0.53	0.65
177	↓ 0.68       B 0.81	0.73       −0.55         0.51        0.51       −0.50       −0.58     −0.57     −0.63          0.52           0.60      −0.59       0.56          −0.56        −0.54        −0.52         0.69       −0.60        −0.50  −0.53      0.54	0.51

178	↑ 0.71      W 0.78	0.71          0.63      −0.59      0.60       0.51       0.53       0.59       0.60      −0.54         0.50       0.58      −0.52         0.60       0.53          0.53      −0.65         0.58       0.56       0.69       0.52    −0.56

179	♀ 0.56      5 0.58      W 0.61	0.66         −0.54        −0.51        −0.53        −0.65        −0.56        −0.51        −0.65         0.51         −0.53        −0.51         0.56         −0.54         0.55          −0.51        −0.53        −0.53        −0.55         0.67       0.50  −0.61
180	♀ 0.59       5 0.56       B 0.55	−0.74         0.52           0.60           0.50         −0.60         0.52           0.54           0.64           0.54           0.57        0.50           0.58           0.57        0.50           0.55        0.53           0.52        0.53     0.54          0.52	0.52
181	♂ 0.58       ↑ 0.65      W 0.58	−0.63         0.54      −0.66     −0.59      0.52       0.51      −0.54         0.55      −0.53     −0.58         0.52      −0.54      0.56        0.51           0.50         −0.64      0.60           0.52          0.56        0.55      −0.61
182	♀ 0.55      ↑ 0.65      W 0.75	−0.55         0.51         −0.54       −0.51       −0.56     −0.51         0.55          0.51      −0.52      0.65          0.56         −0.52         0.52          0.56          0.56        −0.65      0.58          0.55          0.54          0.53       −0.52
183	−0.62         0.57       0.57       0.56      −0.63         0.59          0.54       0.53        0.53          0.54       0.51        0.53         −0.60         0.53           0.51           0.53          0.59        0.50          0.60        0.51      0.56

184	♀ 0.57       5 0.56       B 0.70	0.67       0.60       0.64          0.59       0.50       0.53       0.54      −0.52         0.58          0.55      −0.60      0.61      −0.56      0.57          0.54          0.62       0.50       0.51        0.53      0.50	0.57
185	♂ 0.61       5 0.55       B 0.62	0.59       −0.57         0.61       0.53      −0.62     −0.54        −0.57       −0.52       0.58           0.53       −0.52         0.57       −0.55         0.56         −0.55         0.58       0.50        0.56      0.63       0.55	0.62
186	↑ 0.58       B 0.57	−0.72         0.57       0.58       0.55       0.54       0.54       0.57       0.50        0.56         −0.65         0.54           0.52           0.52           0.55           0.52         −0.55         0.56        0.56      0.57       0.52	0.55

187	↓ 0.61     W 0.60	0.65          0.55       0.67      −0.54       −0.58       −0.54       −0.53       −0.58      −0.58      0.60        −0.51       −0.55       −0.54       −0.51       −0.51      −0.61     0.62      −0.51      −0.59     −0.52       −0.54

188	↓ 0.65      B 0.55	0.68           0.57           0.67           0.57         −0.52       −0.50         0.58      −0.51       −0.55       −0.65       −0.54         0.56         −0.54       −0.56       −0.55      0.57           0.61         −0.53     −0.52      −0.55	0.65

189	♀ 0.60       ↓ 0.65       B 0.69	−0.55       −0.60      0.58       0.58       0.66        0.58          0.54      −0.52         0.55          0.54      −0.58      0.60      −0.53     −0.51        −0.51         0.63       0.55        0.54        −0.55      0.50	0.65
190	↓ 0.74       B 0.61	−0.71     −0.51       0.56        0.56       −0.60      −0.53       −0.50      −0.52       −0.51     −0.63      −0.51          0.57          −0.56        −0.51       −0.52       0.54         −0.62  −0.54       −0.51     −0.51	0.53

191	↓ 0.67       B 0.73	0.58       −0.53         0.58       0.54       −0.52        −0.55         0.58       −0.60         0.56           0.57       −0.54         0.57      −0.56     −0.55        −0.56         0.71       0.54       −0.50  −0.53      0.56	0.64

192	♂ 0.58	W 0.62	0.52  0.60          −0.56        −0.52        −0.66        −0.55        −0.51        −0.56        −0.53         0.66      −0.54        −0.50        −0.51        −0.55        −0.53        −0.52         0.61      −0.51        −0.52       −0.51	0.58
193	5 0.58	0.69           0.55        0.61           0.57        0.50           0.57           0.54        0.63           0.51           0.62           0.52           0.52         −0.64         0.52           0.57           0.52           0.54        0.54         −0.68         0.50    0.57

194	↑ 0.74	−0.58         0.54       0.66       0.51       0.64           0.54           0.55          0.62       0.52       0.53       0.53       0.50          0.60       0.55          0.60      −0.57      0.50       0.58          0.62       0.50         −0.59

195	♀ 0.72      ↓ 0.74	−0.58     −0.52         0.66         −0.50       −0.64     −0.52        −0.52        −0.63         0.57         −0.51        −0.50         0.57       −0.59        −0.58        −0.54         0.51         −0.51        −0.58       −0.52     −0.50  −0.53
196	↑ 0.70	0.68       0.58      −0.66      0.55       0.66       0.51       0.52       0.59       0.52           0.63          0.53       0.54           0.54           0.52           0.54         −0.57        0.58       0.50           0.62           0.55       −0.64

197	♀ 0.57       ↓ 0.70       B 0.82	−0.69     −0.59         0.52        0.54      −0.56       0.60         −0.59     −0.64          0.55           0.57      −0.52      0.61       −0.58        −0.50        −0.53         0.71        0.65       −0.51  −0.53     −0.55	0.58
198	↑ 0.56      B 0.59	−0.55        0.52       0.68          0.50      −0.54       −0.58         0.58          0.54       0.53          0.51       0.58          0.50          0.58          0.52         −0.55         0.53          0.51       0.52    0.53      0.50	0.68

199	↓ 0.62      B 0.66	0.51      −0.51        0.51       0.50        −0.61     −0.53         0.54         −0.59         0.52          0.52      −0.51         0.53         −0.54       −0.57       −0.53        0.67       0.60         −0.50       −0.52       −0.57	0.62
